# Explaining international differences in excess mortality due to Covid-19

**DOI:** 10.1038/s41598-025-92403-z

**Published:** 2025-04-22

**Authors:** Donya Brown, Martina Dattilo, James Rockey

**Affiliations:** 1 Bank of Jamaica, Kingston, Jamaica; 2 Department of Economics and Statistics “Cognetti de Martiis”, University of Turin, Turin, Italy; 3 University of Birmingham, Birmingham, England

**Keywords:** Socioeconomic scenarios, Health policy, Public health

## Abstract

Many explanations have been advanced for why the frequency of deaths associated with Covid-19 varied so much across countries. Previous work has provided evidence that numerous social, economic, and environmental factors correlate with Covid-19 outcomes. One problem researchers face in identifying which of these explanations are best able to explain cross-country variation is that the number of these explanations is too large to be usefully included in a single regression model. This paper uses Bayesian Model Averaging (BMA) to address this problem, focusing on excess mortality to ensure meaningful comparisons across countries. The results suggest that a key determinant of countries’ success in containing Covid-19 has been the strength of the Rule of Law. We also find evidence that rainfall and seaborders are key potential explanations for differences in excess mortality.

## Introduction

With more than 6 million confirmed deaths, the SARS-CoV-2 (Covid-19) pandemic has been the greatest health crisis of the century. “*The suffering and loss we have all endured will be in vain unless we learn the painful lessons from COVID-19*” declared WHO Director-General, Dr Tedros Adhanom Ghebreyesus in September 2022. A key challenge in learning these lessons is explaining countries’ differing success in combating and preventing Covid-19 given that the number of potential explanations is large relative to the number of countries. Moreover, given the novel nature of the pandemic, there is no prior literature on the basis of which to identify a number of candidate statistical models to compare. Instead, we must be agnostic about which combination of factors best explains variation across countries in excess mortality. Thus, this paper uses Bayesian Model Averaging (BMA) techniques, which provide a principled way of addressing such model uncertainty, to disentangle which explanations have the most empirical support when applied to a dataset comprising countries accounting for almost 99% of global GDP.

The key takeaway from this analysis is that the quality of institutions, particularly in terms of the Rule of Law and the control of corruption, are robust predictors of excess mortality rates. Of course, as well as the quality of government varying, so did the complexity of the challenge they faced. The results suggest that — all else equal — countries with more vulnerable populations such as greater incidence of Diabetes or lower prior-exposure to Malaria had higher excess mortality. Perhaps reflecting the greater complexity of dealing with the pandemic in those countries. Likewise, the results suggest that maritime nations fared better, perhaps reflecting the greater ease of imposing quarantine at sea-borders relative to land-borders.

These main results are not altered when we condition on the forms of the Covid-19 policies adopted by governments, suggesting the importance of the quality of policy implementation. 

The variation in countries’ success in preventing the spread of Covid-19, and limiting deaths due to it, is striking. As of the 26th of July 2021, the rate of fatalities per 100, 000 people varied from as much as 600 in Peru to less than 1 in a number of countries including China, New Zealand, and Singapore^1^. Even amongst more obviously comparable countries, there are large differences. The USA’s rate of 186 compares unfavourably to Canada’s of 71. Likewise, the UK and the Netherlands suffered mortality rates of 194 and 110 respectively. More broadly, mortality in many African countries such as Nigeria or Kenya, 1 and 7 respectively, has been lower than elsewhere, and may be seen as particularly so given limited access to vaccines and healthcare. One explanation is that both the definition of a Covid-19 fatality and surveillance capabilities vary considerably across countries. For example, USA and Canada define a Covid-19 death as “clinical diagnosis-based”; while for UK and Netherlands, to define Covid-19 deaths a positive test is required. To address such discrepancies, we study the differences in excess mortality across countries, a more reliable and comparable measure.

The other natural explanation is that countries differed in their exposure to the virus, in their ability to manage its spread, and to treat those who caught it. More generally, when governments come to take stock of the policy lessons from the pandemic, understanding the sources of these differences in Covid-19 related deaths will be an important source of learning. While these questions require a causal analysis, a first step in building understanding is to understand the correlations that exist between excess mortality and underlying factors. Our BMA approach allows us to understand the relative explanatory power of the explanations already proposed in the literature. We focus on 2020 as this allows us to study the correlates of an effective response to a novel disease separate to countries’ ability to obtain and distribute vaccines.

One class of such explanations highlights pre-existing differences between countries such as the sophistication and organization of health-care systems^2,3^ or city size and population-density^4–10^, or climate^11–13^, or indeed the weather^14,15^. A related, but distinct, line of work has focused on air quality^16,17^.

A second has emphasized the role of non-pharmaceutical interventions (NPIs) such as banning large gatherings, stay-at-home orders^18–23^ or mandatory facial coverings^24^. A final class of explanations focuses on differences in government capability and effectiveness^25–32^. That is, differences in governments’ ability to resource, design, and implement effective policy.

If the explanatory variables were all independent of each other then we could evaluate their relationship with Covid-19 outcomes one by one using simple bivariate regression models. However, this is not the case. Moreover, while it is likely that all of these factors have some explanatory power when considered separately, identifying which have explanatory power *conditional* on other important factors is both more relevant and more challenging. But, answering this question, and thus identifying which of these explanations has the most empirical support, is both useful in its own right and as a first step in developing a systematic causal analysis which would address what can, and indeed cannot, be done to combat Covid-19 and future respiratory viruses. For example, evidence that population density or climate were the key determinants of Covid-19 transmission would potentially imply very different government policy to evidence that NPIs were the key factor. However, the surfeit of available explanations and the small ‘population’ of countries with which to study them precludes a standard classical approach in which we include all explanations in an initial model and test down to choose some ‘best’ model on the basis of a given criterion. Our approach also avoids issues of path dependence and overly large models that can affect other approaches^33–35^.

The BMA approach of this paper is a principled way to deal with the twin problems of uncertainty about which is the correct statistical model and a finite sample. BMA treats the underlying population statistical model as something also to be estimated alongside the more conventional regression coefficients and standard errors. To do so it considers the entire *model space*, that is all possible combinations of which covariates to include. By sampling from this model space, it identifies which models have good explanatory power. One need not focus on identifying a single best model, rather we can focus on identifying the variables that often appear in models with high explanatory power. This is done by introducing the Bayesian Adaptive Sampling (BAS) algorithm. This samples models without replacement from the model space, thus yielding marginal inclusion probabilities from which we can draw our inferences about a pool of selected covariates importance as correlates of Covid-19 outcomes^36^. We make use of recent developments in the BMA methods to obtain results that are robust to outliers, and remain stable across small permutations of the data. Additionally, we assess the estimates of the posterior inclusion probabilities from the BMA models under different conditions (with and without predetermined policy controls and regional differences-such as Continents) to garner greater insights into the core predictors of Covid-19 outcomes.

## Methodology

### Dependent variables

We address our research question by using the excess mortality per 1,000 people as our dependent variable. This allows us to bypass differences in reported deaths due to Covid-19 due to differences in definitions, surveillance capabilities, or even political interference, etc.

More precisely, we study differences in excess mortality in 2020 as calculated by the World Mortality Dataset (WMD)^37^ and the World Health Organization (WHO)^38^. The idea is that deviations from the long-run average in 2020 will reflect the aggregate impact of the pandemic. This includes both deaths due directly to Covid-19, regardless of national definitions, and also additional deaths due to cancelled treatments for other conditions, etc. Less intuitively, but importantly, the excess mortality data will also include any positive impacts of the pandemic such as reductions in annual deaths due to flu or even reductions in traffic fatalities.

**Fig. 1:**
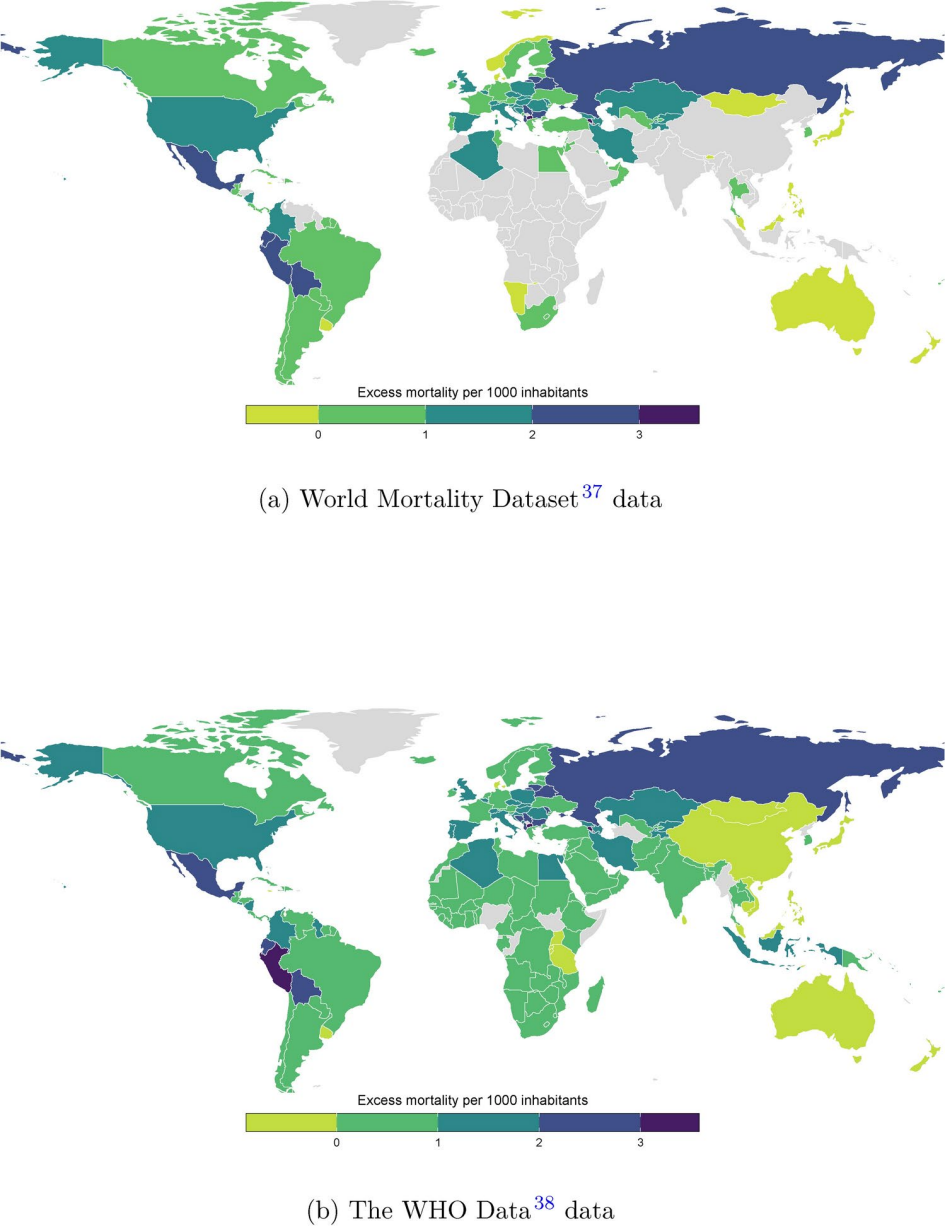
Excess Mortality (year 2020). Source: Created by the authors using RStudio (Version 1.1.456).

Figure 1 maps excess mortality by country for the two data sources^37,38^. The first thing we notice is that excess mortality varies substantially, including between adjacent countries or those we otherwise expect to be similar.

Second, we can see that excess mortality is indeed negative in some countries. This will in part perhaps reflect random annual variation - but may also reflect that in countries with very few Covid-19 deaths restrictions on travel, school closure, shelter-in-place orders, and the comparative paucity of winter-flu deaths meant that aggregate mortality was below its recent average.

Our preferred measure of excess mortality would be the WMD^37^, produced by the leading global research consortium for mortality data. However, as Fig. 1a reveals, their geographical coverage is partial and by excluding much of Africa and Asia, including China and India, much of the world’s population is not covered.

Thus, we work with the WHO data which extends the WMD^37^ to cover over 229 countries, that together account for the vast majority of the world’s population.

These data are mapped in Fig. 1b. We can see that excess mortality is lower in absolute values, but the relative distribution is the same in the Fig. 1a. In addition, in most of the additional countries, it is lower than in Europe or the Americas.

This is as expected, given that Covid-19 infections are more likely to lead to death in older people, and the additional countries mostly have younger populations. However, our BMA approach means that when we come to analyse the predictors of excess mortality we do not make any assumptions about the underlying cause, but include variables that capture age and other sources of vulnerability in the set of candidate variables.

The WHO^38,39^ data is derived using a Bayesian GLM approach to combine the many disparate sources of data that are available for countries not covered by the WMD^37^ to impute excess mortality. As Acosta^40^ writes in their commentary on the *Nature* article introducing the WHO data “*excess mortality [...] is considered the gold-standard approach for estimating the mortality toll of short-term events*”. Nevertheless, they also emphasize the challenges in estimating excess mortality globally – in many cases, particularly in low-income countries, as Fig. 1a makes clear, the necessary data are either imperfect, or in some cases missing altogether. Helleringer^41^ outlines many of the key challenges in measuring excess mortality. Some concerns – e.g. that data are not recorded in a timely fashion – are less of a concern in our context, others such as a lack of data on the age profile, or very little data for some countries at all,^40^ are a very real concern, and our empirical strategy is designed with these shortcomings of the data in mind. Acosta concludes that *“Although the inferences made by Msemburi and colleagues are not ideal, there is no obvious alternative. However speculative these estimates, most are surely closer to the truth than are officially reported numbers of deaths from COVID-19. To rely on confirmed deaths would imply that the pandemic spared low-income and lower-middle-income countries - vulnerable populations that have limited capacity for testing and response. This assumption is highly implausible, and even irresponsible.”*.

**Fig. 2:**
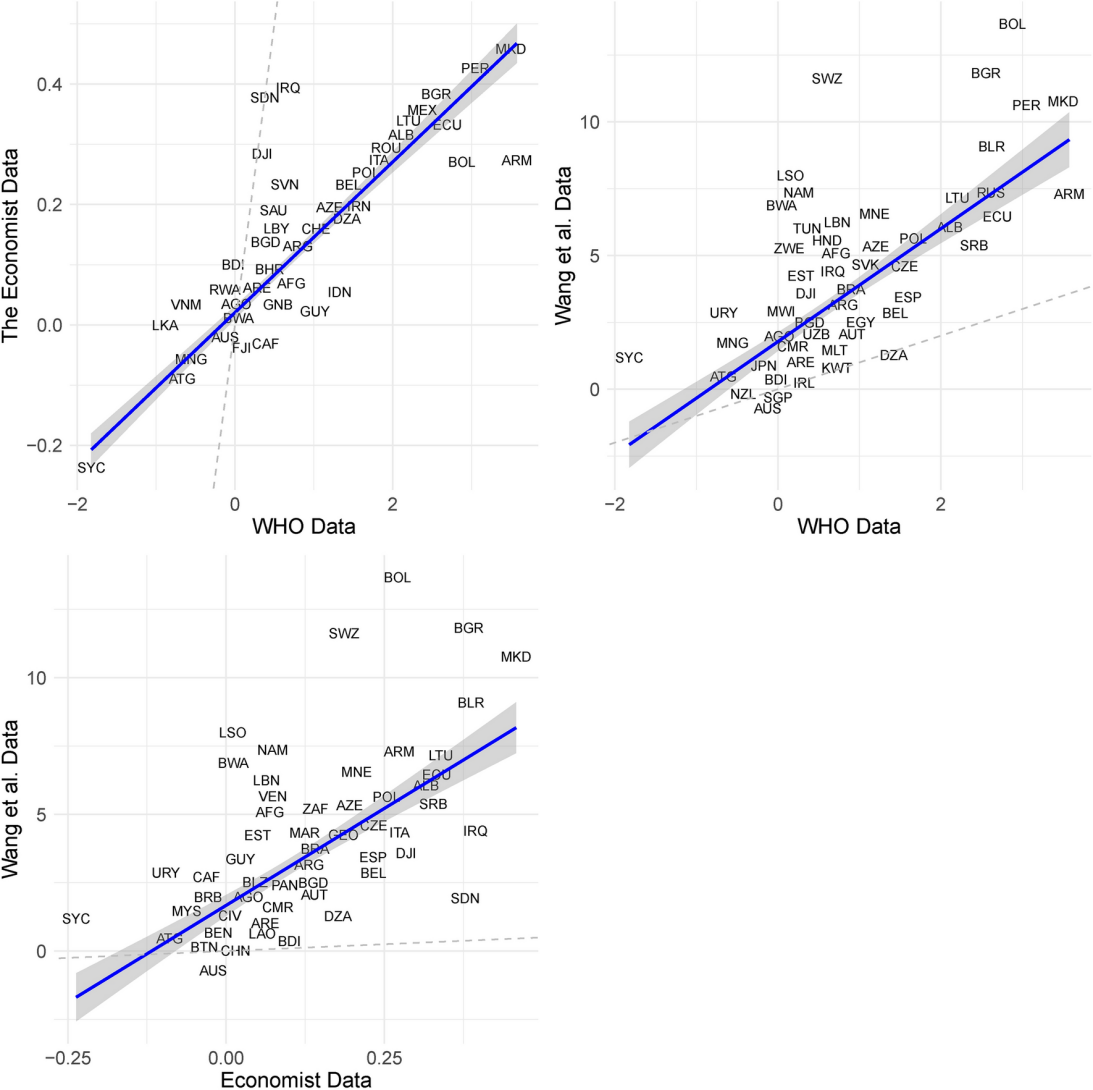
Comparison of the Economist and Wang et al. Datasets versus the WHO Data (year 2020).

Additional reassurance is provided by comparing the WHO data with two alternative datasets compiled by the Economist^42^ or by Wang et al.^43^. In Fig. 2, we report scatter plots of each of the alternative datasets and the WHO data. Looking at the two plots together, three things are immediately clear. First, comparison of the dashed 45° line and the majority of the data points, reveals the WHO estimates, as Acosta^40^ notes, are lower than those of obtained using Machine Learning methods by *The Economist*^42^ or by Wang et al.^43^. Second, most observations are clustered around the line of best fit, reflecting the fact that the rank ordering of excess mortality rates is similar across the three datasets. This is also reflected by the fact that the correlation coefficient of the WHO data with these alternative datasets is relatively high at 0.85 and 0.64 respectively. Third, there are a few outlying observations, particularly in the Wang et al. data, something our empirical strategy is designed to address.

### Independent variables

The independent variables that we consider are chosen on the basis of two criteria. Firstly, we restrict our attention to variables for which data are available for a sufficiently large proportion of countries. For example, the number of ventilators in a country may seem an important factor in predicting the number of deaths, but reliable data are only available for a small set of countries. On the other hand, data on the number of hospital beds or the number of nurses per capita is available for most countries. Moreover, concerns about collinearity preclude including highly correlated variables. Thus, continuing the example, whilst the number of ventilators may contain important additional information relative to our other health variables in some cases, in general, proliferating alternative proxies of the same underlying concept such as healthcare system capacity is not useful.

Secondly, we choose variables on the basis of those factors that have been identified in prior literature. Indeed, most of our variables are included based on the Covid-19 literature discussed above. We also include factors that have been identified in the literature on the determinants of economic growth as good descriptors of differences across countries such as education levels; corruption and the rule of law; trade-openness; and the nature of a countries borders. We break these into seven groups:

1. ***Demographic variables*** including the total population, population density, the urban population share, percentage of people with Diabetes, the sex ratio, average education, average annual deaths per capita due to natural disasters, and the ethno-linguistic fragmentation^44^, population affected by natural disasters, life expectancy, median age;
2. ***Economic variables*** such as GDP per capita, inflation, government indebtedness, the balance of payments, trade openness, percentage of the population with an internet connection, women’s labour market participation, gini coefficient, unemployment, total labour force participation;
3. ***Health system capacity variables*** such as the number of doctors, nurses, and hospital beds as well as health expenditure, governments’ expenditure and the Global Health Security Index (GHS Index), measuring countries’ capacities to prepare for epidemics and pandemics.
4. ***Worldwide Governance Indicators (WGI)*** including *Voice and Accountability*, *Political Stability* and *Absence of Violence/Terrorism*, *Government Effectiveness*, *Regulatory Quality*, *Rule of Law* and *Control of Corruption*.
5. ***Environmental Indicators*** — Given prior work has emphasized both climatic differences and air quality we include measures of average rainfall and CO2 emissions per capita.
6. ***Geographic controls*** — We also include details on the number of land and sea borders (defined as the number of other countries’ exclusive economic zones that intersect a given country’s exclusive economic zone), extracted from the Correlates of War Project (CoW). In addition, we include a set of binary variables for continents and a measure of airlines-connectivity^45^. Finally, we include data on the rates of Malaria infection and mortality, from the Malaria Atlas Project.
7. ***Government Policy Indicators*** — To understand whether differences in outcomes were in part due to differences in policies, our dataset also includes measures of governments’ interventions, sometimes termed NPIs. These are taken from the Oxford Covid-19 Government Response Tracker (OXCGRT)^46^. These data contain daily time-series measures of NPIs including school closures, restrictions on gathering, stay-at-home requirements, contact tracing, or facial covering requirements. They also include measures of governments’ economic support for their citizens. We compute the stringency subindex of each measure, and then we aggregate these indexes over the first year to match our dependent variable.

Each of these variables has itself been much studied in other contexts, and so we will not discuss any of them at length here. However, full details of all the variables we include, their sources, and what transformations were applied are tabulated in Table S.1 of the Supplementary Materials. In addition, the Supplementary Materials also contain a detailed description of how we handle the missing data and the correlation matrix (Figure S. 1).

Our sample covers most countries and the majority of the global population. The countries included are very varied in key characteristics, as confirmed by a brief inspection of the other variables. For example, GDP per capita varies from $0.27 to $107, and the number of physicians varies from 0.04 per thousand people to over 8. A list of the countries included in our data is reported in Table S.4 in the supplementary materials.

In addition, the independent variables considered have different characteristics. Factors such as climate, population density, or government effectiveness change meaningfully during the course of the pandemic. While others, such as government debt or unemployment have changed over time, and indeed due to the pandemic, then we will focus on the correlation with their pre-Pandemic values. Our approach will thus be cross-sectional — analysing what explains differences in countries’ success in combating Covid-19 during the first year of the pandemic. This approach is distinct from, and complementary to, approaches in epidemiology^47–49^ that seek to explicitly model the spread of Covid-19 in the population and the impact of NPIs on that spread.

### Bayesian model averaging

The aim of this paper is to understand the cross-sectional variation in countries’ experience of the pandemic.

This rich diversity in outcomes, national characteristics and policy presents an important opportunity to identify risk factors and policies most correlated with lower excess mortality. However, the large number of candidate explanations enumerated above is in contrast to the number of ‘cases’, here countries.

In such settings, Bayesian Model Averaging (BMA) has much to offer. The fundamental difference between other approaches and that of BMA, is that BMA, regards different empirical models as being drawn from a distribution of possible models. Instead of the traditional approach of attempting to identify the best or most plausible model to describe a relationship, BMA assumes that any “true” model is extremely unlikely to be identified by the available data. Instead, it allows the investigator to identify which models are more or less plausible, given the data and the prior distribution over models. That is, it provides a principled framework to deal with the uncertainty about which factors are important for explaining the variation in pandemic outcomes in the face of limited data, namely a finite number of countries to sample.

BMA estimators have been developed for a range of regression models^50^ but here, for simplicity of inference and computational reasons, we focus on linear regression with normally distributed standard errors with the additional assumptions that the model is additive in its variables, and we take variable transformations as given. The following explanation follows that of Clyde et al.^36^ closely. The *p* potential explanatory variables are denoted $\varvec{X_{1}, X_{2},\ldots , X_{p}}$. A given model $\varvec{\mathcal {M}_{\gamma }}$ is described in terms of which explanatory variables it includes by a vector of binary variables $\gamma =\left( \gamma _{1},\ldots ,\gamma {p}\right) ^{\prime }\in \left\{ 0,1\right\} ^{p}\equiv \Gamma$ such that $\gamma _{j}$ is an indicator of whether $\varvec{X_{j}}$ is in $\varvec{\mathcal {M}_{\gamma }}$. (Properly, included as a column in the $n \times p_{\gamma }$ design matrix $\varvec{X_{g}}$ for model $\varvec{\mathcal {M}_{\gamma }}$.) Then we can write the individual regression for model $\varvec{\mathcal {M}_{\gamma }}$ analogously to a conventional regression model, as:

$$\begin{aligned} \varvec{Y}\mid \alpha ,\varvec{\beta _{\gamma }},\sigma ^{2},\varvec{\mathcal {M}_{\gamma }}\sim N\left( \mathbb {1}\alpha +\varvec{X_{\gamma }\beta _{\gamma }}, \varvec{I_{n}} \sigma ^{2}\right) \end{aligned}$$.

Where $\varvec{Y}=\left( y_{1},\ldots ,y_{n}\right)$, $\mathbb {1}$ is a vector of ones of length *n*, $\alpha$ is the intercept, $\varvec{\beta _{\gamma }}$ are the regression coefficients, and $\sigma ^{2}$ is the error variance. This says that the distribution of $\varvec{Y}$, here our measures of Covid outcomes, conditional on a given model $\varvec{\mathcal {M}_{\gamma }}$ and its associated regression coefficients $\varvec{\theta _{\gamma }}=\left( \alpha ,\varvec{\beta _{\gamma }},\sigma ^{2}\right)$ is normally distributed with mean $\alpha +\varvec{X_{\gamma }\beta _{\gamma }}$ and variance $\sigma ^{2}$. Thus, it is differentiated from the standard definition of a linear regression model only by the inclusion of $\varvec{\mathcal {M}_{\gamma }}$ and the model subscripts on $\varvec{X_{\gamma }}$ and $\varvec{\beta _{\gamma }}$.

Contra, model or variable selection procedures BMA approaches use the full joint-posterior distribution (the posterior distribution over models and coefficients) to incorporate uncertainty about which (if any) of the candidate models is the ‘right’ or ‘true’ model.

Following Hoeting et al.^51^ and Clyde et al.^36^, for a given quantity of interest, $\Lambda$, such as a particular regression coefficient, we can calculate its posterior distribution over the set of models $\varvec{\mathcal {M}}$ given the dataset *Y* as:

1 $$\begin{aligned} pr(\Lambda \mid Y)=\sum \limits _{\varvec{\gamma } \in \Gamma } pr(\Lambda \mid \varvec{\mathcal {M}_{\gamma }},Y)pr(\varvec{\mathcal {M}_{\gamma }}\mid Y) \end{aligned}$$

Equation (1) states that the probability of a given value of $\Lambda$ given the data is equal to the probability of that value of $\Lambda$ given the model and the data multiplied by the posterior probability of that model given the data. Similarly, we can compute the expectation of $\Lambda$ taken over all models as:

2 $$\begin{aligned} E\left[ \Lambda \mid \varvec{Y}\right] =\sum \limits _{\varvec{\gamma } \in \Gamma } E\left[ \Lambda \mid \varvec{\mathcal {M}_{\gamma }}, \varvec{Y}\right] pr(\varvec{\mathcal {M}_{\gamma }}\mid \varvec{Y}) \end{aligned}$$

To understand what Eq. (1) describes it is useful to consider the constituent terms in more detail. Again following Hoeting et al.^51^ the posterior probability of any given model $\varvec{\mathcal {M}_{\gamma }}$ is:

3 $$\begin{aligned} pr(\varvec{\mathcal {M}_{\gamma }}\mid \varvec{Y})=\frac{pr(\varvec{Y} \mid \varvec{\mathcal {M}_{\gamma }})pr(\varvec{\mathcal {M}_{\gamma }})}{\sum \limits _{\varvec{\gamma } \in \Gamma }pr(\varvec{Y} \mid \varvec{\mathcal {M}_{\gamma }})pr(\varvec{\mathcal {M}_{\gamma }})} \end{aligned}$$

Finally, they define the following:

4 $$\begin{aligned} pr(\varvec{Y} \mid \varvec{\mathcal {M}_{\gamma }})=\int pr(\varvec{Y} \mid \varvec{\theta _{\gamma }},\varvec{\mathcal {M}_{\gamma }})pr(\varvec{\theta _{\gamma }}\mid \varvec{\mathcal {M}_{\gamma }})\,d\varvec{\theta _{\gamma }} \end{aligned}$$

Loosely, Eq. (4) can be thought of as the posterior probability distribution of observing the data given a particular model $\varvec{\mathcal {M}_{\gamma }}$. Hence, Eq. ( 3) defines the posterior probability distribution of a given model $\varvec{\mathcal {M}_{\gamma }}$ as the chance of observing the data given that model multiplied by the prior probability of that model divided by the sum of the same product for every model. Finally, Eq. (1) represents the posterior probability distribution of $\Lambda$ for a particular model conditional on the observed data multiplied by the posterior probability of that model given the data, summed across all models.

BMA requires the researcher to make two choices^52^. Firstly, the prior distribution of the regression coefficients in each model $\theta _{\varvec{\gamma }}$. Secondly, the prior probability of each possible model. For the first we use the hyper-g priors^53^. This involves assigning the g-prior of Zellner^54^ to the regression coefficients:

5 $$\begin{aligned} pr\left( \alpha , \varvec{\beta _{\gamma }} \mid \varvec{\mathcal {M}_{\gamma }}\right) \propto \sigma ^{-1} f_{N}^{p_{\gamma }}(\varvec{\beta _{j}} \mid 0, \sigma ^{2} g\left( \varvec{Z_{\gamma }^{\prime }Z_{\gamma }}\right) ^{-1} \end{aligned}$$

Where $f^{p_\gamma }_{N}$ is the density function of a $p_\gamma$ dimensional Normal distribution. Thus, this prior distribution for each regression coefficient has a mean of zero and a precision in proportion to the variance of the associated variable. The choice of $g>0$ determines how informative the prior is assumed to be. For example, $g=n$ then the prior is assumed to have the same informational content as a single observation. In general, the choice of *g* will have a substantial influence on the results^52^, and thus we introduce a hyper-prior on *g* (hence the name hyper-g) requiring the choice of a single parameter *a*. Following the relevant literature^36,52^, we choose $a=3$.

Our choice of prior over the model space is slightly different from the prior literature. In particular, we employ a more informative prior over the expected model size. Early BMA estimators required the researcher to choose the probability $\phi$ with which each regressor is included in a given model. For example, Raftery et al.^55^ assumed all regressors have a prior inclusion probability of 0.5. Subsequently, Ley & Steel^56^ proposed putting a hyper-prior on $\phi$, thus treating it as an additional random variable to be estimated. Following Ley & Steel^52^, we assume a beta-binomial(1,6) hyper-prior.

We are however concerned about the potential for overly large models, termed the supermodel effect^57^. Thus, we also report results using a Poisson(1) prior truncated at 20 variables. That is we assign prior probability of 0 to all models with 21 or more regressors. Whilst, somewhat *ad hoc* this choice is designed to more heavily-penalise very large models.

## Results

This section presents the main results of the Bayesian Model Averaging (BMA) analysis. Our final dataset includes 162 countries representing 95% of the global population and 99% of global GDP. Table 1 contains summary statistics for each of the main variables included in the analysis: the dependent variable, i.e. Excess Mortality per capita (EM), and the independent variables including demographic, economic, health system capacity variables, the Worldwide Governance Indicators, environmental indicators and geographic controls. Table 2 reports the results from four different specifications of the model.

**Table 1:**
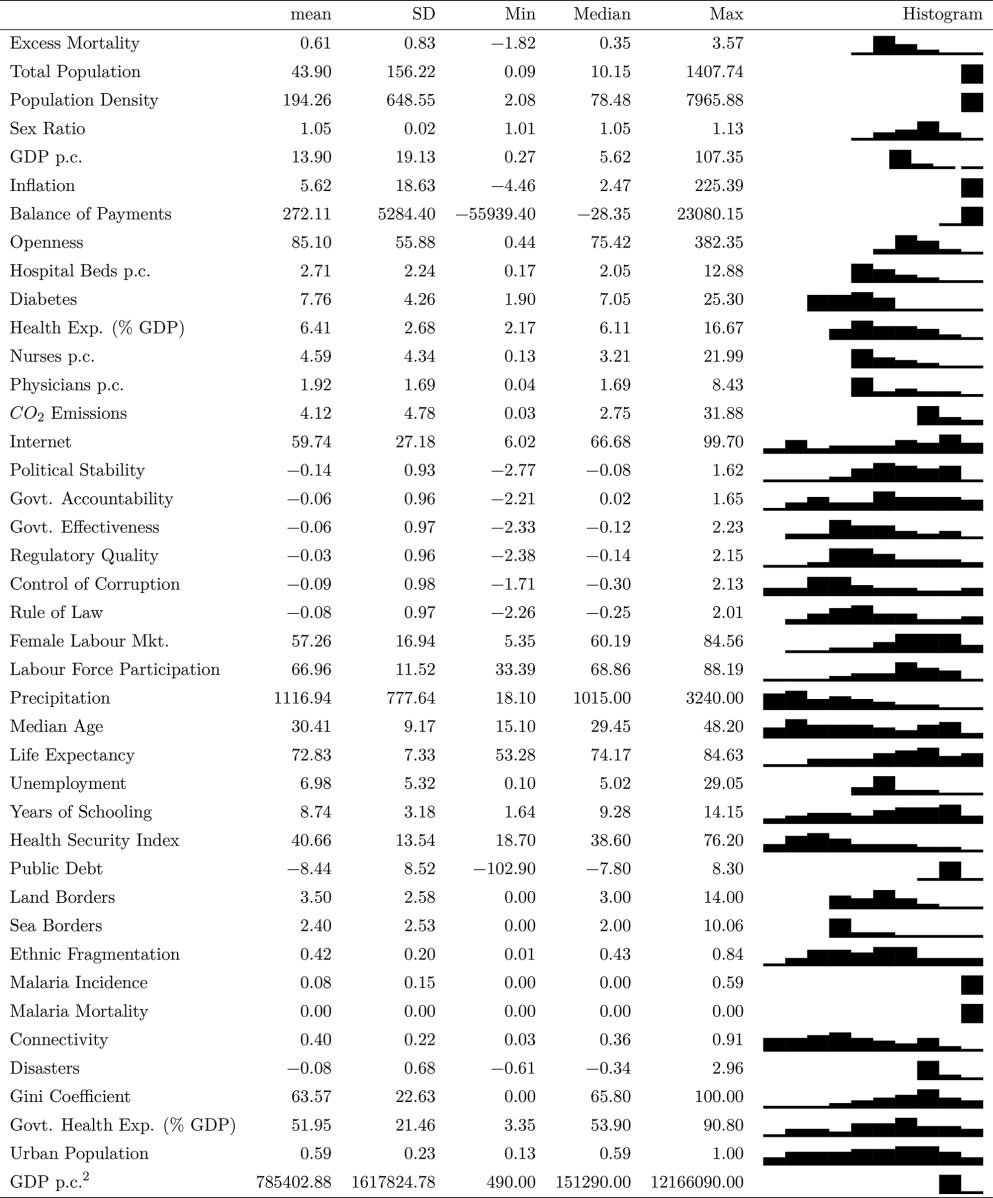
Summary statistics

**Table 2:**
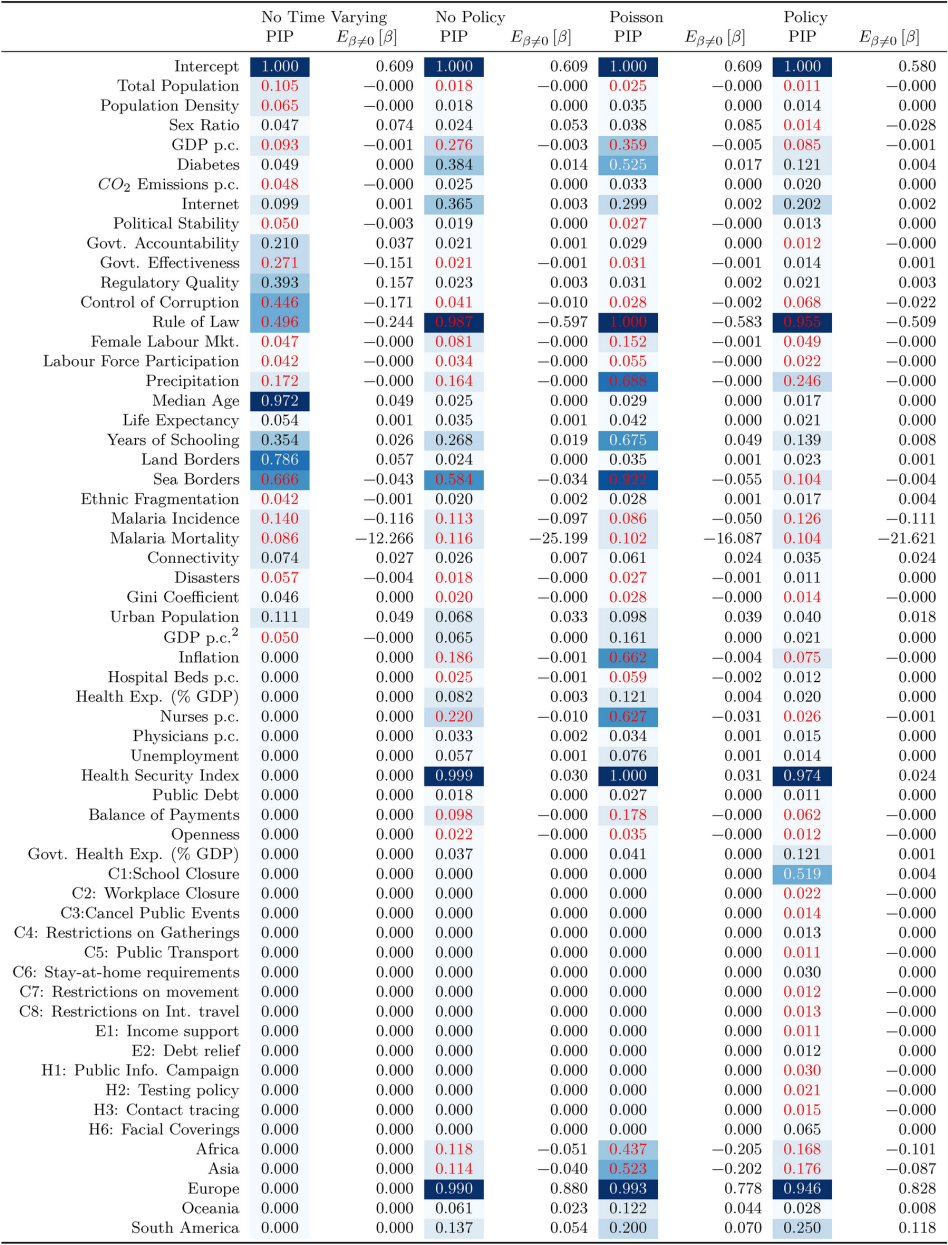
Correlates of cross-country differences in excess mortality (WHO data). Each column reports the results of a separate Bayesian Model Averaging analysis. The coefficients in columns 1,3,4, and 5 are Posterior Inclusion Probabilities (PIPs). That is, $\left( Pr(\beta \ne 0 | \varvec{Y}\right)$, the sum of the posterior probabilities of models that variable appears in. The dependent variable is excess mortality in the first year of the Covid-19 pandemic. See section "Methodology" for further details. Estimates were obtained using the BAS package of Clyde (2011)^36^ assuming a hyper-g/n prior^53^ over coefficients and a beta-binomial(1,6) prior over the model space, except for columns 5 and 6 which use a truncated-Poisson(1,20) prior. See section. "Bayesian model averaging" for further details.

Many of the variables in our dataset affect or depend on each other; thus, to build our intuition, we first consider only *pre-determined* variables. That is, those that are either fixed, or change only slowly over time and are thus less amenable to policy. The results of the specification excluding time-varying variables and the Covid-19 policy variables are reported in the first two columns of table 2. Two key statistics are reported for each regressor: the posterior inclusion probability (PIP) (first column), as defined in Eq. (3), and the posterior conditional expected coefficient (second column), as given in Eq. (2). An interpretation of the latter is that it is the (model-probability weighted) average coefficient on variable *j* across those models that include *j*. To emphasize this interpretation, we henceforth denote it as $E_{\beta \ne 0}\left[ \beta \right]$. For clarity, the PIPs of variables for which the estimated coefficients are negative are reported in red font. Likewise, variables with a higher PIP have a darker background, and vice versa. We plot the PIPs in Fig. 4.

Reading down the first two columns of table 2, we see that the largest PIP (excluding the intercept) is for the median age of the population with a PIP of 0.97 and a positive coefficient. This is strong evidence that, consistent with what we expect, excess mortality was higher in countries with older populations, other things equal. $E_{\beta \ne 0}\left[ \beta \right]$ is 0.049 which implies, that a population in which the median individual is 10 years older is expected to have additional excess mortality of around 0.5 per 1,000 people. This is a large effect in both absolute terms, but also in terms of the cross-country variation in Covid-19 related excess mortality, where it is equivalent to an increase of 0.6 standard deviations. We also see a PIP of 0.35 on the average years of schooling completed in the population, with $E_{\beta \ne 0}\left[ \beta \right] =0.026$. This suggests that more educated populations, other things equal, had higher excess mortality rates. Given that we do not expect education to directly increase Covid-19 related excess mortality this coefficient presumably is proxying for other aspects of better-educated societies such as other features of the age distribution, the type of jobs, urbanization, income levels, etc.

Next, we see that both the Land Borders and Sea Borders variables have high PIPs of 0.79 and 0.67, respectively, suggesting that a larger number of land border or sea borders is an important predictor of excess mortality. The coefficient for Land Border is positive, suggesting that countries with a great number of adjacent countries had, other things equal, higher levels of excess mortality. Further analysis would be necessary to establish the causal mechanisms, but one explanation is that countries with a greater number of land-borders makes it more difficult to control the spread of Covid-19, and vice versa for sea borders.

Finally, there is a group of variables capturing various aspects of high-quality institutions. The Rule of Law has the highest PIP at just under one half, followed by Control of Corruption (0.45), Regulatory Quality (0.39), Government Effectiveness (0.27), and Government Accountability (0.21). The $E_{\beta \ne 0}\left[ \beta \right]$ estimates imply substantial effects of these variables. For example, they suggest that a country with a one standard deviation higher Rule of Law index should be expected to have 0.24 fewer excess deaths per 1,000 people. Taken together, they imply that a country’s quality of government is a first-order predictor of its excess mortality rate. Note, that

Figure 4 reports the same results as in columns one and two of table 2. The upper plot reports the PIPs of each variable, with the variables with a positive conditional expected coefficient coloured green, and those with a negative coefficient coloured red. Those variables which have a PIP of 0.5 or above, make up the Median Probability Model. Whilst, we prefer to base our inference on the average across all possible models, the Median Probability Model is a guide to what specific models are judged likely by the BMA procedure, and thus provides some comparability with conventional frequentist analyses. We can see that the Median Probability Model is extremely parsimonious, it includes only the median age, land borders and sea borders (narrowly excluding the Rule of Law). Our analysis is Bayesian and thus not focused on statistical significance. However, we can consider a variable as ’significant’ if its PIP is greater than chance, i.e., the prior expected model size divided by the number of explanatory variables (here 12), $\frac{6}{29}=0.21$^58^.

The lower panel of Fig. 4 provides a description of the posterior model space. At the bottom of the plot, we see the Highest Probability Model, which includes the Control of Corruption, median age, Land Borders, and Sea Borders only. Additional models are plotted above it, with their height proportional to their posterior probability. The left-hand y-axis reports the cumulative posterior probability of the top *n* models, allowing us to assess the overall uncertainty about the true model^51^. Here, we can see that the single most likely model has a posterior model probability of 2.1%, and the top 10 models have a total posterior model probability of 12.2%. Given that there are $2^{29}$ or 530 million possible models, we interpret this as suggesting that the BMA approach is performing relatively well at identifying the key predictors.

As well as describing the models with the highest posterior probability, they also help us understand the overall composition of models, rather than considering each variable in isolation, as if it were orthogonal to the other candidate variables^59,60^. We can see that median age is included in many of the top ranked models, as are land and sea borders. Interestingly, Control of Corruption and the Rule of Law variables seem to substitute for each other. Again we see that the top ranked models are all relatively parsimonious. Our interpretation is twofold. First, the results suggest that the BMA procedure is identifying small models that capture each of the different factors discussed above. Second, and importantly from a statistical point of view, it is evidence that there is not a problem with the supermodel effect^57^.

**Fig. 3:**
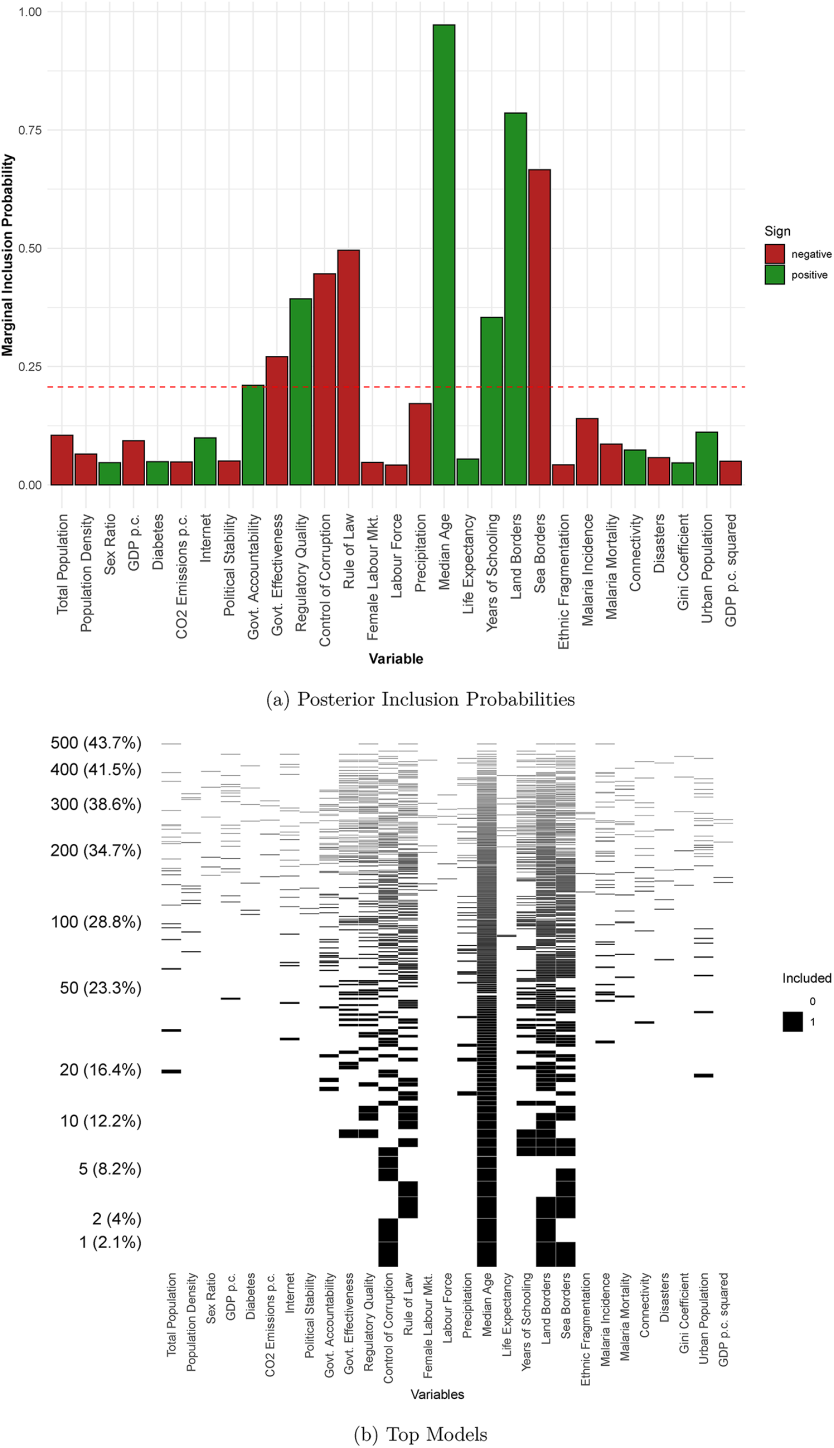
Determinants of cross-country differences in excess mortality: excluding policy.

The next two columns of Table 2 and Fig. 3 report results extending the set of regressors to include time-varying variables as well as continent dummies. The latter are designed to capture consistent differences across continents. Indeed, different continents were affected at different times, and the countries within them may have other institutional or climatic features in common. We can immediately see that the European continent dummy has a PIP close to 1 and a posterior coefficient of 0.88. This suggests that European countries had consistently higher excess mortality rates than might otherwise have been expected. The coefficients on Asia and Africa are both negative, suggesting the opposite pattern in those countries, but with PIPs only just above chance levels.

We no longer find evidence that the number of land borders is a good predictor of excess mortality, but the PIP on sea borders is still high (0.58). Likewise, the PIP of the Rule of Law is now close to 1 with an increase in the magnitude of $E_{\beta \ne 0}\left[ \beta \right]$ to $-0.6$, but this is at the expense of the other government quality variables.

Unsurprising, perhaps, is that the PIP of the Global Health Security Index which is almost identically 1. More surprising, is that the posterior conditional expected coefficient is positive. Given we would not expect countries scored to be better able to prepare for, and combat, a pandemic, to have higher excess mortality, this might suggest that the causal mechanism runs the other way. That is, it is the countries most at risk that are also those that are best able to prepare. Establishing, such a causal claim would be an important topic for future research. Although the PIP is smaller, we likewise find a positive coefficient on Health Expenditure as a percentage of GDP.

Other variables describing the health system are easier to interpret. The coefficient on the number of nurses per capita is negative with a PIP of 0.219. The coefficient suggests that, other things equal, countries with 10 additional nurses per 1,000 people, had excess mortality rates 0.1 deaths per 1,000 lower. This is a strong relationship, and it is interesting that we find no evidence that the number of physicians per 1,000 people is a good predictor of excess mortality. Nor do we find evidence for a (partial) correlation with the number of hospital beds per capita, government spending on healthcare, or overall spending on healthcare.

Whilst, life expectancy is a good measure of the overall health outcomes in a population it may not capture that some populations are at greater risk of dying due to Covid-19 than others. We include the incidence of Diabetes in the population since there is evidence^61^ that people living with Diabetes are at greater risk of severe illness due to Covid-19, as are those living with common comorbidities of Diabetes^61^. The PIP is 0.38 and the posterior conditional expected coefficient is 0.014 implying that, other things equal, a 10% higher incidence of Diabetes is an increase in excess mortality of 0.14 deaths per 1,000 people. Relatedly, we also include the incidence of Malaria and mortality due to Malaria as potential controls since it has been suggested that prior-exposure to Malaria may offer some protection from Covid-19^62–64^. It may also be the case that countries that are experienced in dealing with infectious diseases have more effective responses than those that are not. Our estimates suggest some support for this hypothesis, although both variables have a PIP only slightly higher than chance levels the coefficients are quite large. For example, the coefficient on Malaria Mortality implies that countries where the Mortality rate is 1 death per 1000 people higher due to Malaria would have an excess mortality rate of 0.25 lower. We find no evidence of a correlation with experience of natural disasters. Finally, we find that the share of the population with Internet access is positively correlated with excess mortality. Again, this seems unlikely to be a direct effect. Inspection of the data suggests that the countries where Internet access is least common are predominantly in sub-Saharan Africa, and as such it may be that Internet access is proxying for the lower rates of Covid-19 mortality observed in low-income, less urbanized countries.

As we might expect, richer countries, other things equal, seem to have lower excess deaths due to Covid-19. The PIP on GDP per capita is now 0.28, although the relationship is not terribly strong: the coefficient implies that an additional $\$10,000$ per year is associated with 0.03 fewer deaths. However, the results suggest no impact of our other measures of macroeconomic performance — unemployment, inflation and public debt. Likewise, income inequality as captured by the Gini coefficient, does not seem to be a good predictor of excess mortality. There is some evidence that a Balance of Payments surplus is negatively related to excess mortality, but the PIP is not above chance levels.

There is some evidence that higher average rainfall is associated with lower excess mortality, with a PIP of 0.16. This result is consistent with what is now a large body of literature finding a correlation between rainfall (or other climatic factors such as sunlight^65^) and Covid-19 spread or mortality. However, we note that our finding is about the correlation with average rainfall at the national level, and not the correlation between high-frequency weather changes within a country as in some other work. The explanation for this correlation remains unclear. One class of explanations is that rain alters behaviour in such a way that reduces excess mortality, such as staying at home^14,15^; another is that rainfall somehow directly affects the spread or danger of Covid-19^12^; another is that rainfall is correlated with some unmeasured factors predicting excess mortality causing the correlation with rainfall to be spurious.

Looking at the top models in panel (b) of Fig. 3 suggests that as above the BMA approach seems to be successfully identifying a subset of models with relatively high posterior model probabilities. The probability of the single most likely model is 1.4% and that of the top 10 models is 8.5%, which is large relative to the space of $2^{45}$, or 35 trillion, candidate models.

Columns 5 and 6 report results using an alternative model prior. Instead of the beta-binomial prior recommended by Ley & Steel^52^, we instead use the truncated Poisson distribution with mean one, and a maximum of 20 included variables. Using this prior will favour models with relatively few regressors, and the truncated prior will rule out the ‘supermodel effect’^57^ in which a few (overly) large models dominate the posterior model space. We see that while the exact PIPs and mean coefficients vary a little, the results are otherwise very similar. This suggests that a few overly large models are not driving our results.

**Fig. 4:**
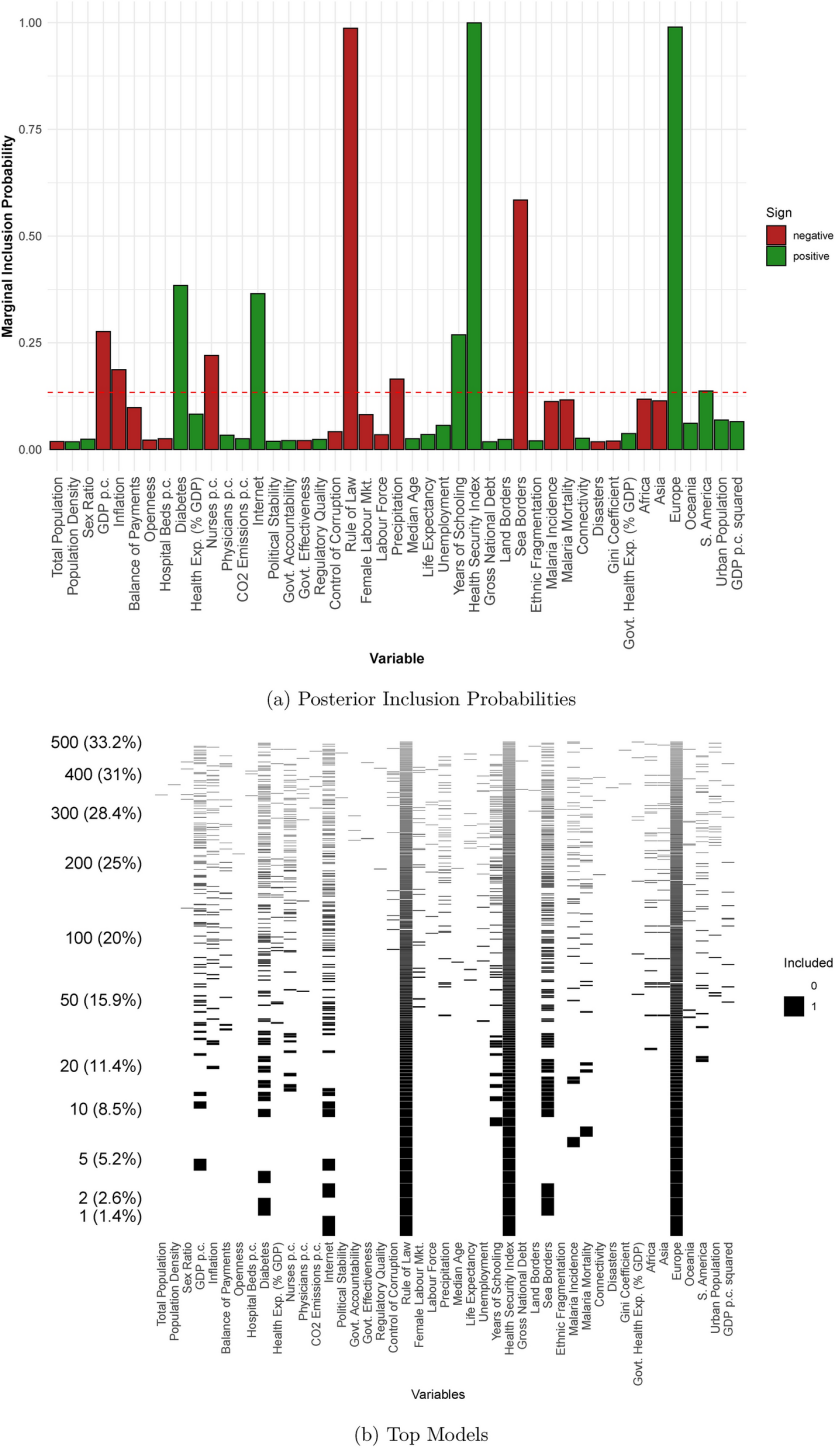
Determinants of cross-country differences in excess mortality: pre-determined covariates.

### Mechanisms

Taken together our results suggest that the key sources in variation in excess mortality were factors that are associated with the health and age of the population such as the median age, the number of nurses, and the incidence of communicable and non-communicable diseases such as Diabetes and Malaria. The relationship with development is complicated and nuanced. While, higher per capita incomes are negatively correlated with excess mortality, outcomes were worse, other things equal, in Europe than in other continents, and the least developed countries, those in which Internet access is still rare, also had lower excess mortality rates. One very clear finding was that, other things equal, the Rule of Law seems to be an important predictor of outcomes. This raises the question of why the quality of institutions was important. One possibility is that more capable governments enacted different policies, another is that differences in implementation were more important.

**Fig. 5:**
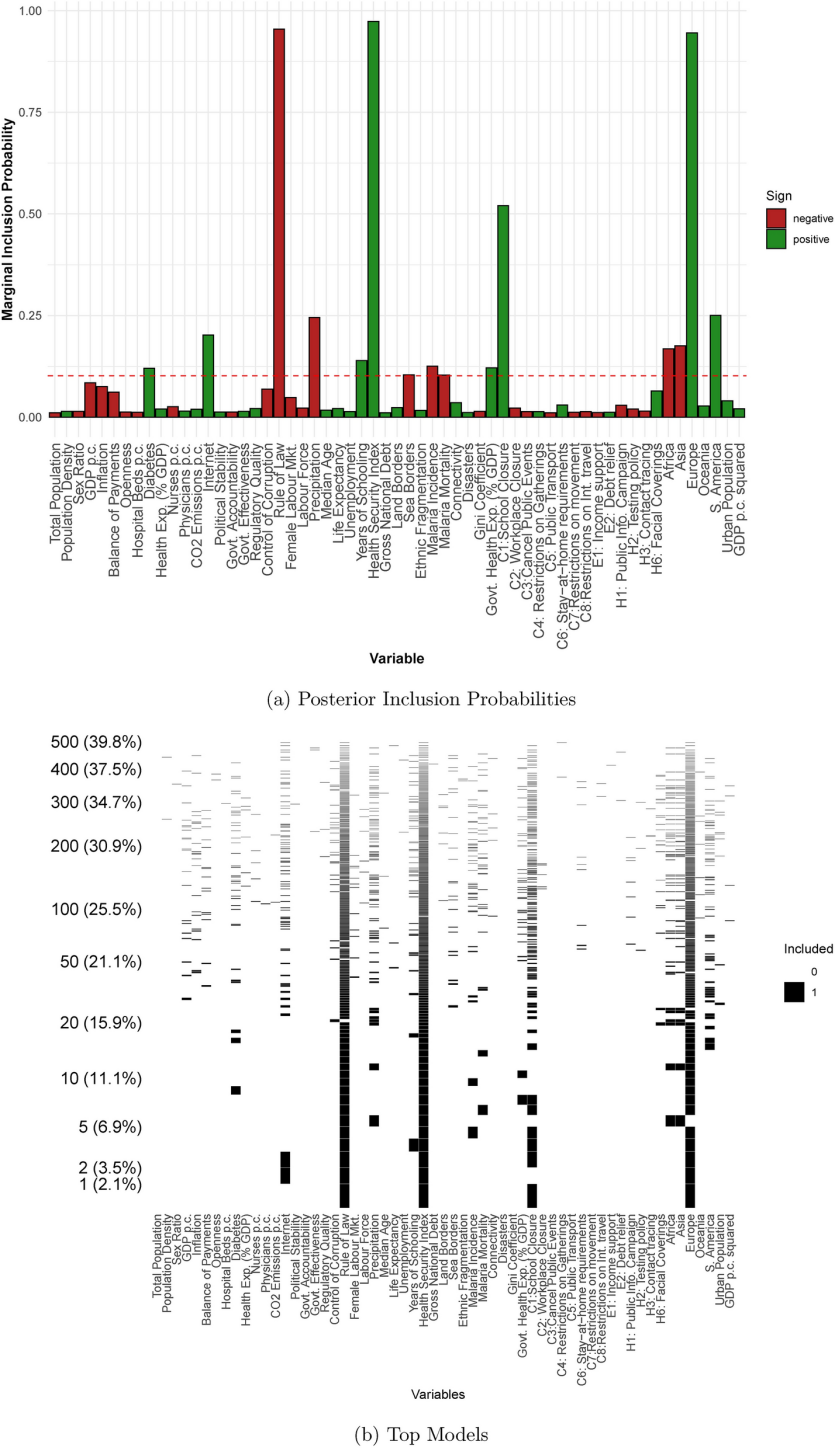
Determinants of cross-country differences in excess mortality: all covariates.

To assess this, we additionally include the OXCGRT policy measures (see the Supplementary information for the summary statistics). We aggregate these policies as the average over the period covered by the dependent variable. These estimates, reported in columns 7 and 8 of table 2 and in Fig. 5, are not easy to interpret directly, as they themselves will be endogenous outcomes of the prior number of deaths and the expected number of future deaths.

The first policy measure, C1: School Closures, is positively correlated with excess mortality with a PIP of 0.52. It seems likely that this is because countries struggling most to manage the pandemic were more likely to have to close schools, rather than school closures somehow driving excess mortality.

None of the other policy variables have PIPs in excess of chance levels. Indeed, they are all extremely close to zero. This need not imply that the policies were unimportant. Importantly, the results for other variables are largely unaffected. Some PIPs are smaller, but this is to be expected given there are now more candidate variables for any model of a given size, and we do not change the model prior. If differences in national characteristics had given rise to different policies and this was why there were differences in excess mortality, then we would expect that these different policies were likely to replace those characteristics in the more likely models. Given that they do not, then there is reason to believe that countries in which the Rule of Law was stronger had better policy implementation. That is, we continue to find a correlation between the pre-determined country characteristics and excess mortality whilst potentially allowing for endogenous policy choice, suggesting that the correlation between characteristics and excess mortality is not due to relationships between those characteristics and policy choice.

That we don’t find effects of most policies may also reflect the limitations of our approach. It is plausible, for example, that our analysis finds conflates countries in which they were implemented early and prevented excess deaths, and places where they were implemented only after excess mortality was already high. Table 3 reports OLS estimates of the five models with the highest posterior probabilities. These help clarify the structure of the most likely models, and allow for conventional inference. We report the $R^{2}$ alongside other statistics. The $R^{2}$ suggests that the relatively parsimonious models reported are able to explain nearly half of the total variation in excess mortality.

**Table 3:** Linear regression for the top 5 models.

	Dependent variable: Excess Mortality per 1,000				
	(1)	(2)	(3)	(4)	(5)
Internet Access			$$0.009^{***}$$		$$0.007^{***}$$
			(0.002)		(0.002)
Rule of Law	$$-0.557^{***}$$	$$-0.494^{***}$$	$$-0.708^{***}$$	$$-0.516^{***}$$	$$-0.600^{***}$$
	(0.086)	(0.079)	(0.103)	(0.078)	(0.092)
Average Years of Schooling	$$0.061^{***}$$				
	(0.021)				
Health Security Index	$$0.023^{***}$$	$$0.028^{***}$$	$$0.029^{***}$$	$$0.025^{***}$$	$$0.024^{***}$$
	(0.007)	(0.007)	(0.008)	(0.006)	(0.007)
Malaria Incidence				$$-0.925^{***}$$	
				(0.227)	
School Closures	$$0.009^{***}$$	$$0.010^{***}$$		$$0.009^{***}$$	$$0.008^{***}$$
	(0.003)	(0.003)		(0.003)	(0.003)
Europe	$$0.901^{***}$$	$$1.003^{***}$$	$$0.856^{***}$$	$$0.973^{***}$$	$$0.955^{***}$$
	(0.145)	(0.148)	(0.160)	(0.146)	(0.145)
Constant	$$-1.840^{***}$$	$$-1.576^{***}$$	$$-1.352^{***}$$	$$-1.293^{***}$$	$$-1.735^{***}$$
	(0.333)	(0.326)	(0.313)	(0.333)	(0.328)
Observations	162	162	168	162	162
$\hbox {R}^{2}$	0.467	0.440	0.438	0.467	0.469
Adjusted $\hbox {R}^{2}$	0.450	0.426	0.424	0.449	0.452
Residual Std. Error	0.570	0.583	0.629	0.570	0.569
F Statistic	$$27.361^{***}$$	$$30.817^{***}$$	$$31.729^{***}$$	$$27.292^{***}$$	$$27.507^{***}$$
$$\delta$$	0.15	$$0.024^{**}$$	0.17	0.14	0.16

The Table presents linear regression estimates of the 5 models with the highest posterior probabilities in the BMA analysis. $\delta$ results from Ramsey’s RESET test for functional form accounting for the second and third order. The statistic is only statistically significant in column 2 implying that for the other top models we can not reject the null-hypothesis of linearity.

Significance: $$^{*}$$p<0.1, $$^{**}$$p<0.05, $$^{***}$$p<0.01. Robust standard errors in parentheses.

We also report the results of a RESET test of our assumption of a linear functional form. The statistic is only statistically significant in column 2 implying that for the other top models we can not reject the null-hypothesis of linearity. Whilst, only suggestive, this reduces concerns that our assumption of linearity is overly restrictive.

## Discussion and concluding remarks

In order to better understand Covid-19 and to guide policy, a substantial body of work has sought to identify the key social, economic, and environmental factors which determine its spread. This paper has used Bayesian Model Averaging (BMA) techniques to assess which of the explanations proposed are best able to explain cross-country variation in excess mortality due to Covid-19.

The results suggest that a key determinant of countries’ success in containing Covid-19 has been the strength of the Rule of Law. Countries in which corruption is controlled, and citizens have more confidence in the rules of society performed considerably better than those in which they are not. Some of our other findings may be viewed as capturing the difficulty faced by governments. Some populations began the pandemic more vulnerable in terms of demographics and other risk factors than others. Likewise, others potentially had additional protection, for example, due to prior exposure to Malaria. Of course, these findings may partly reflect the period we study, when Covid-19 mortality rates were highest and much was not yet understood about the treatment and transmission of Covid-19.

Which variables our analysis suggests are not robust correlates of excess mortality is also interesting. We find no role for $CO_{2}$ emissions, or population density, despite previous support for them in the literature. Even more surprisingly, perhaps, is that in our analysis we find evidence of a reversal-of-fortunes effect in which greater healthcare system capacity is associated with higher mortality rates. Controlling for Covid-19 policy responses did not alter our results. Our interpretation of these findings is that which policies were implemented was less important than how effectively they were used.

One limitation of our study is that we have studied excess mortality over the period as a whole. In future work, it would be extremely worthwhile to develop methods and suitable excess mortality data which would allow us to understand how different national characteristics mattered at different points of the pandemic, and potentially the impact of policy on excess mortality thus bridging the gap with much of the other literature that studies the impact of particular policy interventions. Likewise, some of the robust correlations we document do not yet have a clear or intuitive explanation, beyond reverse causality, such as that on the Health Security Index. We hope that our findings can serve as a guide to productive avenues for further research that can better map the causal pathways. Doing so may reveal, for example, greater impacts of the policy variables we consider. We have largely excluded candidate variables that are highly collinear with those we include, such as alternative measures of GDP, or more detailed demographic controls. It would be interesting for future research to employ dilution priors on the model space^66–68^ to address this collinearity and allow for the distinction between highly correlated variables.

## Supplementary Information

Supplementary Information.

## Data Availability

All the data used for this study are publicly available; all the details regarding the original data sources employed, including the web links, are reported in the Supplementary Materials.
