# Supplementary material for: Explaining international differences in excess mortality due to Covid-19
Source: Sci Rep. 2025 Apr 22;15:13879. doi: 10.1038/s41598-025-92403-z (PMC12015526; doi:10.1038/s41598-025-92403-z)
Supplement: Supplementary file 1 — Supplementary Information. [file 41598_2025_92403_MOESM1_ESM.pdf]

Explaining International Differences in Excess Mortality due to Covid-19

Donya Brown[1] Martina Dattilo [2 *] James Rockey[3]

1 - Bank of Jamaica 2 - University of Turin 3 - University of Birmingham

31st December 2024

Supplementary Information

Excess Mortality Data

As discussed in the data section, we used *excess mortality*$_i$, as our dependent variable. It records the estimated excess deaths associated with COVID-19 pandemic from all-causes per 100,000 in country i in 2020. This measure is obtained from the WHO Data [1]. The procedure WHO followed to produce excess mortality estimates consisted of three steps: estimating a provisional number of excess deaths associated with the COVID-19 pandemic, consulting WHO member states on the estimates and incorporating countries' feedback into the modelling to update the estimates. The data used for the prediction come from multiple sources: countries' self-reported information (spontaneous or following WHO data call), Eurostat and other national statistics offices (such that of China, Grenada, Saint Kitts and Nevis, Saint Vincent and the Grenadines, Sri Lanka and Vietnam), the Human Mortality Database [2;3] and the World Mortality dataset [4]. Annual historic mortality data was used to forecast the expected all-causes mortality to 2020. For all countries and time points, they model the expected numbers on the basis of historical data using a negative binomial model that allows for excess-Poisson variation. Finally, the excess mortality estimates are obtained by using a conventional statistical modelling approach in which a parametric model is fitted using Bayesian inferential machinery.

Explanatory variables

As mentioned in the Methods section, we select a number of explanatory variables which can be grouped into the following dimensions: Demographic variables, Economic variables, Health system capacity variables, Worldwide Governance Indicators (WGI), Environmental Indicators, Geographic controls, Government Policy Indicators. Table S.1 details the variables included in each category and shows the data source. Most of the explanatory variables are extracted from the World Bank Open Data using the World Bank API.

The daily sub-indexes of the stringency index for each policy are calculated as: $100 \times \frac{(v_{j,t} - 0.5(F_j - f_{j,t})}{N_j}$, where $v_{j,t}$ is the recorded policy value on the ordinal scale, F_j is equal to 1 if the indicator has a flag variable and 0 otherwise, $f_{j,t}$ the recorded binary flag for this indicator, N_j the maximum value of the indicator and t corresponds to one day. The flag indicator differentiates two levels of implementation, distinguishing if a policy is implemented at the national or local level. As a consequence, the stringency index formula allows us to attribute a higher value to the policy implemented at the national level. Our final indicators represent the average of the daily sub-indexes in the year 2020.

Each policy's theoretical minimum and maximum value are reported in Table S.2; while descriptive statistics are reported in Table S.3.

Imputation of missing data

To reduce reverse causality issues, we extract the explanatory variables referring to the year 2019. When an observation is not available, earlier years (starting from 2005) are used to proxy the 2019 values. For the majority of the variables considered, the proportion of countries for which the 2019 observation is missing is negligible. There are two cases that are worth mentioning. First, the variable *Hospital Beds p.c.* has a high number of missing observations in 2019, resulting in a significant difference between the mean and the median year of extraction (average year: 2014.5; median year: 2017). In addition, the most recent observation period for the variable *Diabetes* is 2011, *Precipitation* was observed in 2017, and *Disaster* in 2009.

For some variables, there was a significant lack of countries observed, but a second reliable and comparable source was available. In the case where only a few information were missing, we impute missing data with information from secondary sources. Then, we remove the countries with more than 20% missing observations. This choice resulted in the exclusion of the following countries: Andorra, Cook Island, Dominica, Micronesia, Saint Kitts and Nevis, Monaco, Marshall Island, Niue, Nauru, Palau, DPR Korea, San Marino, Sao Tome and Principe, Tuvalu. We also remove the countries that have missing values for variables which did not have any second source available, such as *Connectivity*, or *Ethnic Fractionalisation*, resulting in the removal of the following countries: Congo, Grenada, Maldives, South Sudan, Saint Lucia (LCA), Saint Vincent and the Grenadines, Brunei Darussalam. Myanmar, Nigeria, Equatorial Guinea and Turkmenistan.

Table S .1: Variable Definitions and Sources

Demographic variables

Variable Name	Description	Data Source
Total Population	Total population	**World Bank API** Code: SP.POP.TOTL
Population Density	People per sq. km of land area	**World Bank API** Code: "EN.POP.DNST"
Sex Ratio	Male births per female births (5 year averages)	**World Bank API** Code: "SP.POP.BRTH.MF"
Urban Population	Percentage of urban population	**World Bank API** Code: "SP.URB.TOTL"
Diabetes	Comorbodities like diabetes prevalence (% of population ages 20 to 79)	**World Bank API** Code: "SH.STA.DIAB.ZS"
Years of Schooling	Mean years of schooling	**Human Development Reports** Source: https://hdr.undp.org/
Disasters	Annual average percentage of the population that is affected by natural disasters classified as either droughts, floods, or extreme temperature events. (average 1990-2009)	**World Bank API** Code: "EN.CLC.MDAT.ZS"
Ethnic Fragmentation	Index aggregating Ethnic, Language and Religion fractionalization indexes.	Source: Alesina et al.[5]
Life Expectancy	Life Expectancy	OWID Life Expectancy[6]
Median Age	Median Age	OWID https://ourworldindata.org

Economic variables		
GDP p.c.	GDP per capita (constant 2010 US$)	**World Bank API** Code: "NY.GDP.PCAP.KD"
Inflation	Inflation, GDP deflator (annual %)	**World Bank API** Code: "NY.GDP.DEFL.KD.ZG"
Public Debt	Total stock of debt liabilities issued by the general government as a share of GDP	**International Monetary Fund** Source: https://www.imf.org/ and **World Bank API** Code: "GC.NLD.TOTL.GD.ZS"
Balance of Payments	Net Trade in Goods and Services (BoP current US$)	**World Bank API** Code: "BN.GSR.GNFS.CD"
Openness	Sum of exports and imports of goods and services measured as a share of gross domestic product.	**World Bank API** Code: "NE.TRD.GNFS.ZS"
Internet	Individuals using the Internet (% of population)	**World Bank API** Code: "IT.NET.USER.ZS"
Female Labour Mkt.	Labor force participation rate, female (% of female population ages 15+) (modeled ILO estimate)	**World Bank API** Code: "SL.TLF.ACTI.FE.ZS"
Gini Coefficient	Gini Coefficient (Economic Inequality)	**GHS Index** Source:https://www.ghsindex.org
Unemployment	Unemployment, total (% of total labor force) (modeled ILO estimate)	**World Bank API** Code: "SL.UEM.TOTL.ZS" and ILO https://ilostat.ilo.org/
Labour Force Participation	Labour force participation rate (% of the total working population ages 15+)	**World Bank API** Code: "SL.TLF.CACT.NE.ZS" and "SL.TLF.ACTI.ZS"

Health system capacity variables		
Hospital Beds p.c.	Hospital beds (per 1,000 people)	**World Bank API** Code: "SH.MED.BEDS.ZS"
Health Exp. (% GDP)	Current health expenditure (% of GDP)	**World Bank API** Code: "SH.XPD.CHEX.GD.ZS"
Govt. Health Exp. (% GDP)	Domestic general government health expenditure (% of current health expenditure)	**World Bank API** Code: "SH.XPD.GHED.CH.ZS"
Nurses p.c.	Total number of nurses and midwives per 1000 people	**World Bank API** Code: "SH.MED.NUMW.P3"
Physician p.c.	Total number of physicians per 1000 people	**World Bank API** Code: "SH.MED.PHYS.ZS"
Health Security Index	The Global Health Security Index measures the capacities to prepare for epidemics. It is based on 37 indicators belonging to 6 categories: prevention, detection, rapid response, health system, compliance with international norms, risk environment.	**GHS Index** Source:https://www.ghsindex.org

Worldwide Governance Indicators (WGI)		
Political Stability	It measures perceptions of the likelihood of political instability and/or politically-motivated violence, including terrorism.	**World Bank API** Code: "PV.EST"
Govt. Accountability	It reflects perceptions of the extent to which a country's citizens are able to participate in selecting their government, as well as freedom of expression, freedom of association, and a free media.	**World Bank API** Code: "VA.EST"
Govt. Effectiveness	It reflects perceptions of the quality of public services, the quality of the civil service and the degree of its independence from political pressures, the quality of policy formulation and implementation, and the credibility of the government's commitment to such policies.	**World Bank API** Code: "GE.EST"
Regulatory Quality	It reflects perceptions of the ability of the government to formulate and implement sound policies and regulations that permit and promote private sector development.	**World Bank API** Code: "RQ.EST"
Control of Corruption	It reflects perceptions of the extent to which public power is exercised for private gain, including both petty and grand forms of corruption, as well as "capture" of the state by elites and private interests.	**World Bank API** Code: "CC.EST"
Rule of Law	It reflects perceptions of the extent to which agents have confidence in and abide by the rules of society, and in particular the quality of contract enforcement, property rights, the police, and the courts, as well as the likelihood of crime and violence.	**World Bank API** Code: "RL.EST"

	Environmental Indicators	
Precipitation (*precip*)	Average Precipitation in depth (mm per yr)	**World Bank API** Code: "AG.LND.PRCP.MM" and Climate Knowledge Portal
CO_2 Emissions	CO2 emissions (metric tons per capita)	**World Bank API** Code: "EN.ATM.CO2E.PC"
	Geographic controls	
Sea Borders	Water contiguity is based on whether a straight line of no more 400 miles can be drawn between a point on the border of one state, across open water (uninterrupted by the territory of a third state), to the closest point on the homeland territory of another state. This is designed to capture the number of states with which that country's 200 mile exclusive economic zone could potentially intersect.	**Correlates of War** https://correlatesofwar.org
Land Borders	Land contiguity is defined as the intersection of the homeland territory of the two states in the dyad, either through a land boundary or a river.	**Correlates of War** https://correlatesofwar.org
Connectivity	Normalised index of air-line connectivity. It is computed using a network percolation model on global airline passenger data.	Source Meslé et al.[7].
Malaria Incidence	Plasmodium falciparum and Plasmodium vivax combined incidence rate at year 2000	**Malaria Atlas Project** `https://malariaatlas.org/`
Malaria Mortality	Plasmodium falciparum mortality rate per 100k people at year 2000	**Malaria Atlas Project** `https://malariaatlas.org/`

Government Policy Indicators

C1	School closing (average stringency sub-index)	**OXCGRT** https://covidtracker.bsg.ox.ac.uk/
C2	Workplace closing (average stringency sub-index)	**OXCGRT** https://covidtracker.bsg.ox.ac.uk/
C3	Cancel public events (average stringency sub-index)	**OXCGRT** https://covidtracker.bsg.ox.ac.uk/
C4	Restrictions on gathering size (average stringency sub-index)	**OXCGRT** https://covidtracker.bsg.ox.ac.uk/
C5	Close public transport (average stringency sub-index))	**OXCGRT** https://covidtracker.bsg.ox.ac.uk/
C6	Stay-at-home requirements (average stringency sub-index)	**OXCGRT** https://covidtracker.bsg.ox.ac.uk/
C7	Restrictions on internal movement (average stringency sub-index)	**OXCGRT** https://covidtracker.bsg.ox.ac.uk/
C8	Restrictions on international travel (average stringency sub-index)	**OXCGRT** https://covidtracker.bsg.ox.ac.uk/
E1	Income support (average stringency sub-index)	**OXCGRT** https://covidtracker.bsg.ox.ac.uk/
E2	Debt/contract relief for households (average stringency sub-index)	**OXCGRT** https://covidtracker.bsg.ox.ac.uk/
H1	Public information campaign (average stringency sub-index)	**OXCGRT** https://covidtracker.bsg.ox.ac.uk/
H2	Testing policy (average stringency sub-index)	**OXCGRT** https://covidtracker.bsg.ox.ac.uk/
H3	Contact tracing (average stringency sub-index)	**OXCGRT** https://covidtracker.bsg.ox.ac.uk/
H6	Facial coverings (average stringency sub-index)	**OXCGRT** https://covidtracker.bsg.ox.ac.uk/

Table S .2: Policy Indicators - Description

Policy	Measurement
School closing	From 0 (no measure) to 3 (require closing)
Workplace closing	From 0 (no measure) to 3 (require closing)
Cancel public events	from 0 (no measure) to 2 (require cancelling)
Restrictions on gathering size	From 0 (no restrictions) to 4 (restrictions on gathering of 10 people or less)
Close public transport	From 0 (no measure) to 2 (require closing)
Stay-at-home requirements	From 0 (no measure) to 3 (not leaving the house)
Restrictions on internal movement	From 0 (no measures) to 2 (restrictions in place)
Restrictions on international travel	From 0 (no restrictions) to 4 (total border closure)
Income support	From 0 (no income support) to 2 (more than 50% of salary is replaced)
Debt/contract relief for households	From 0 (no debt relief) to 2 (broad debt relief)
Public information campaign	From 0 (no information campaign) to 2 (coordinated public information campaign)
Testing policy	From 0 (no testing policy) to 3 (open public testing)
Contact tracing	From 0 (no contact testing) to 2 (comprehensive contact tracing)
Facial coverings	From 0 (no policy) to 4 (required outside the home)

Table S .3: Policy Indicators - Summary Statistics

Statistic	N	Mean	St. Dev.	Min	Pctl(25)	Pctl(75)	Max
C1	151	74.355	17.338	0.000	63.021	86.514	100.000
C2	151	59.050	16.289	0.000	51.339	69.444	88.123
C3	151	85.302	17.862	0.000	76.352	98.607	100.000
C4	151	77.356	19.142	0	71.3	88.0	100
C5	151	52.719	27.179	0.000	50.000	71.431	100.000
C6	151	48.291	16.421	0	38.9	60.0	100
C7	151	72.287	22.636	0.000	61.993	85.357	100.000
C8	151	61.124	13.620	0.000	54.474	68.818	89.003
E1	151	46.898	29.520	0	25	74.4	100
E2	151	44.603	26.622	0.000	25.137	69.809	83.607
H1	151	95.069	11.852	0.000	95.780	100.000	100.000
H2	151	49.063	17.533	0.000	34.062	61.111	87.614
H3	151	60.726	23.482	0.000	42.555	80.464	100.000
H6	151	65.904	19.347	0.000	53.733	75.500	100.000

Table S .4: List of Countries and ISO Codes

#	Country	iso3c	#	Country	iso3c	#	Country	iso3c
1	Afghanistan	AFG	2	Angola	AGO	3	Albania	ALB
4	United Arab Emirates	ARE	5	Argentina	ARG	6	Australia	AUS
7	Austria	AUT	8	Azerbaijan	AZE	9	Burundi	BDI
10	Belgium	BEL	11	Benin	BEN	12	Burkina Faso	BFA
13	Bangladesh	BGD	14	Bulgaria	BGR	15	Bahrain	BHR
16	Bahamas	BHS	17	Bosnia and Herzegovina	BIH	18	Belarus	BLR
19	Belize	BLZ	20	Bolivia (Plurinational State of)	BOL	21	Brazil	BRA
22	Barbados	BRB	23	Bhutan	BTN	24	Botswana	BWA
25	Central African Republic	CAF	26	Canada	CAN	27	Switzerland	CHE
28	Chile	CHL	29	China	CHN	30	Côte d'Ivoire	CIV
31	Cameroon	CMR	32	Democratic Republic of the Congo	COD	33	Colombia	COL
34	Comoros	COM	35	Cabo Verde	CPV	36	Costa Rica	CRI
37	Cuba	CUB	38	Cyprus	CYP	39	Czechia	CZE
40	Germany	DEU	41	Djibouti	DJI	42	Denmark	DNK
43	Dominican Republic	DOM	44	Algeria	DZA	45	Ecuador	ECU
46	Egypt	EGY	47	Eritrea	ERI	48	Spain	ESP
49	Estonia	EST	50	Ethiopia	ETH	51	Finland	FIN
52	Fiji	FJI	53	France	FRA	54	Gabon	GAB
55	United Kingdom	GBR	56	Georgia	GEO	57	Ghana	GHA
58	Guinea	GIN	59	Gambia	GMB	60	Greece	GRC
61	Guatemala	GTM	62	Guyana	GUY	63	Honduras	HND
64	Croatia	HRV	65	Haiti	HTI	66	Hungary	HUN
67	Indonesia	IDN	68	India	IND	69	Ireland	IRL
70	Iran (Islamic Republic of)	IRN	71	Iraq	IRQ	72	Iceland	ISL
73	Israel	ISR	74	Italy	ITA	75	Jamaica	JAM
76	Jordan	JOR	77	Japan	JPN	78	Kazakhstan	KAZ
79	Kenya	KEN	80	Kyrgyzstan	KGZ	81	Cambodia	KHM
82	Kiribati	KIR	83	Republic of Korea	KOR	84	Kuwait	KWT
85	Lao People's Democratic Republic	LAO	86	Lebanon	LBN	87	Liberia	LBR
88	Libya	LBY	89	Sri Lanka	LKA	90	Lesotho	LSO
91	Lithuania	LTU	92	Luxembourg	LUX	93	Latvia	LVA
94	Morocco	MAR	95	Republic of Moldova	MDA	96	Madagascar	MDG
97	Mexico	MEX	98	Mali	MLI	99	Malta	MLT
100	Mongolia	MNG	101	Mozambique	MOZ	102	Mauritania	MRT
103	Mauritius	MUS	104	Malawi	MWI	105	Malaysia	MYS
106	Namibia	NAM	107	Niger	NER	108	Nicaragua	NIC
109	Netherlands	NLD	110	Norway	NOR	111	Nepal	NPL
112	New Zealand	NZL	113	Oman	OMN	114	Pakistan	PAK
115	Panama	PAN	116	Peru	PER	117	Philippines	PHL
118	Papua New Guinea	PNG	119	Poland	POL	120	Portugal	PRT
121	Paraguay	PRY	122	Qatar	QAT	123	Romania	ROU
124	Russian Federation	RUS	125	Rwanda	RWA	126	Saudi Arabia	SAU
127	Sudan	SDN	128	Senegal	SEN	129	Singapore	SGP
130	Solomon Islands	SLB	131	Sierra Leone	SLE	132	El Salvador	SLV
133	Serbia	SRB	134	Suriname	SUR	135	Slovakia	SVK
136	Slovenia	SVN	137	Sweden	SWE	138	Eswatini	SWZ
139	Seychelles	SYC	140	Syrian Arab Republic	SYR	141	Chad	TCD
142	Togo	TGO	143	Thailand	THA	144	Tajikistan	TJK
145	Timor-Leste	TLS	146	Tonga	TON	147	Trinidad and Tobago	TTO
148	Tunisia	TUN	149	Türkiye	TUR	150	United Republic of Tanzania	TZA
151	Uganda	UGA	152	Ukraine	UKR	153	Uruguay	URY
154	United States of America	USA	155	Uzbekistan	UZB	156	Venezuela (Bolivarian Republic of)	VEN
157	Viet Nam	VNM	158	Vanuatu	VUT	159	Yemen	YEM
160	South Africa	ZAF	161	Zambia	ZMB	162	Zimbabwe	ZWE

Figure S .1: Correlation Coefficient of the main variables

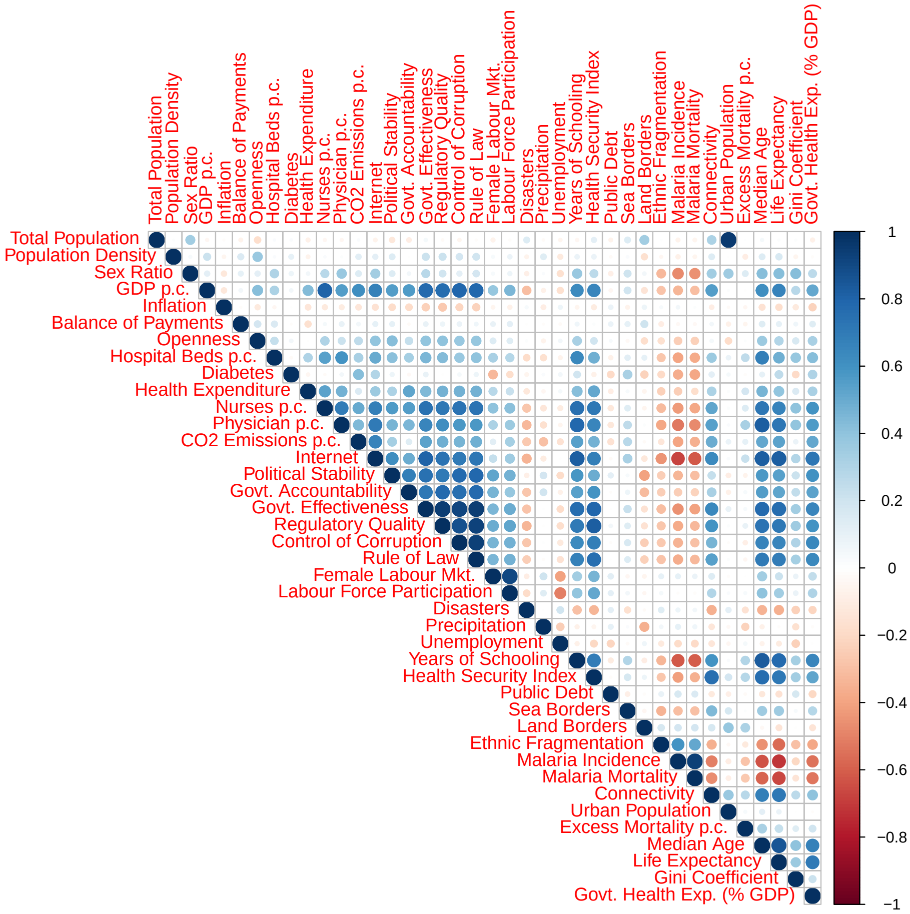

References

[1] Msemburi, W. *et al.* The WHO estimates of excess mortality associated with the COVID-19 pandemic. *Nature* **613**, 130–137 (2023). URL https://doi.org/10.1038/s41586-022-05522-2.

[2] for Demographic Research, M. P. I. & Berkeley, U. C. Human mortality database. short-term mortality fluctuation data series.

[3] Islam, N. *et al.* Excess deaths associated with covid-19 pandemic in 2020: age and sex disaggregated time series analysis in 29 high income countries. *bmj* **373** (2021).

[4] Karlinsky, A. & Kobak, D. Tracking excess mortality across countries during the covid-19 pandemic with the world mortality dataset. *eLife* **10**, e69336 (2021). URL https://doi.org/10.7554/eLife.69336.

[5] Alesina, A., Devleeschauwer, A., Easterly, W., Kurlat, S. & Wacziarg, R. Fractionalization. *Journal of Economic Growth* **8**, 155–194 (2003).

[6] Dattani, S., Rodés-Guirao, L., Ritchie, H., Ortiz-Ospina, E. & Roser, M. Life expectancy. *Our World in Data* (2023). Https://ourworldindata.org/life-expectancy.

[7] Meslé, M. M. I. *et al.* Estimating the potential for global dissemination of pandemic pathogens using the global airline network and healthcare development indices. *Scientific Reports* **12**, 3070 (2022).
